# High screening coverage and positivity rates of HIV, syphilis, and hepatitis B among pregnant women accessing antenatal care in Sierra Leone

**DOI:** 10.5588/pha.25.0051

**Published:** 2026-08-24

**Authors:** M.A. Sesay, B.D. Fofanah, D. Nair, A. Ramsay, N. Sesay, M.S. Kapuwa, I.F. Kamara, M. Sillah-Kanu, I.S. Turay, M. Mustapha, A.M. Falama, W.K. Lahai, F. Kanu, J.A. Koroma, G.N. Kamara, F. Thomas, S. Batcho, S. Lakoh

**Affiliations:** 1 Ministry of Health, Directorate of Disease Prevention and Control, Freetown, Sierra Leone;; 2 World Health Organization Country Office, Freetown, Sierra Leone;; 3 Centre for Operational Research, International Union Against Tuberculosis and Lung Disease, Paris, France;; 4 Division of Infection and Global Health, St Andrews University Medical School, Scotland, UK;; 5 College of Medicine and Allied Health Sciences, University of Sierra Leone, Freetown, Sierra Leone;; 6 KYM Consultancy Ltd. Freetown, Sierra Leone;; 7 34 Military Hospital, Defense Medical Services- Republic of Sierra Leone Armed Forces, Freetown, Sierra Leone;; 8 Department of Pharmacovigilance and Clinical Trials, Pharmacy Board of Sierra Leone, Sierra Leone;; 9 Division of Infectious Disease, Africa Centre for Disease Control and Prevention, Addis Ababa, Ethiopia.

**Keywords:** service coverage, UHC, maternal and child health, primary health care, global fund

## Abstract

**BACKGROUND:**

Sierra Leone faces high rates of mother-to-child transmission (MTCT) of HIV, syphilis, and presumed hepatitis B (HepB). Since 2014, integrated testing and treatment within antenatal care (ANC) have aimed to prevent this transmission, yet no comprehensive study on these infections among pregnant women exists.

**OBJECTIVES:**

Assess screening coverage and test positivity for these infections in 21 health facilities in 4 districts, each representing Sierra Leone’s four regions, to ensure national representation of triple elimination testing: Primary (12), Secondary (5), and Tertiary (4).

**METHOD:**

A cross-sectional analysis of secondary data from health-facility registers in Western Area Urban, Bombali, Bo, and Kenema districts, between November 2024 and October 2025, summarised as frequencies and proportions.

**RESULTS:**

Among 10,003 women attending their first ANC visit, screening coverage was 99.6% for HIV, 99.6% for syphilis, 92.9% for HepB, and 92.4% for all three infections. Test positivity was 4.9% for HIV, 1.3% for syphilis, and 4.0% for HepB.

**CONCLUSION:**

Triple-disease testing at ANC facilities in Sierra Leone is feasible and can achieve high coverage. The test-positivity rate for HepB suggests a considerable burden of maternal infection and ongoing MTCT. Still, there are currently no interventions to address this gap. Testing should be scaled-up urgently and followed by a programme for maternal treatment, safe delivery, and immunisation.

In 2023, approximately 39 million people were living with HIV, 12 million with syphilis, and 300 million with hepatitis B (HepB) worldwide.^1,2^ Vertical transmission of HIV, syphilis, and HepB remains a significant challenge, indicating undiagnosed or untreated infection among pregnant women and high rates of infections in children.^3^ Without effective interventions, the risk of mother-to-child transmission (MTCT) is substantial, with transmission rates of 20%–45% for HIV, 69%–80% for syphilis, and over 90% for HepB.^3^ The sub-Saharan Africa region bears a disproportionately high burden of MTCT of HIV (25.6 million), syphilis (564,000), and HepB (60 million) compared to the rest of the world.^3,4^

The WHO launched the triple elimination strategy (TES) in 2017^5^ to reduce vertical transmission of HIV, syphilis, and HepB through an integrated approach within maternal and child health services.^6–8^ Early detection during antenatal care (ANC), timely treatment, and/or immunisation can prevent MTCT and ensure the mother is diagnosed/treated.^9–11^ The global target is to eliminate these three infections as public health threats among this population by 2030.^12,13^ This initiative is considered highly cost-effective and efficient, especially in low-resource settings.^6^ Although the TES is recommended by the WHO globally^3,6^ and is being adopted by some countries, unfortunately, many sub-Saharan African countries have yet to adopt this initiative.^3,4^ There is very limited evidence on implementation progress, with none from the African region. Reports from the Netherlands,^14^ Indonesia,^15^ India,^16^ and a systematic review from the Pacific Islands^17^ have documented suboptimal implementation.

Sierra Leone faces significant challenges posed by high rates of MTCT of HIV, syphilis, and, it is presumed, HepB.^18–20^ Testing and treatment for both HIV and syphilis have been integrated into ANC services since 2014 to prevent MTCT. The Ministry of Health (MoH) has introduced HepB testing, integrating it with HIV and syphilis testing at ANC facilities in four regional headquarters towns in November 2024, and launched a national TES in February 2025. Testing for all three diseases is conducted as part of the TES supported by the Global Fund programmes. Globally, information on the prevalence of all three infections in pregnant women or on the implementation of TES remains limited. While few studies have primarily focused on one specific aspect of the triple disease, no single study has examined all three diseases among pregnant women, nor the implementation of the new triple testing intervention in Sierra Leone.

This study aimed to assess screening coverage and test positivity for HIV, syphilis, and HepB among pregnant women attending ANC in selected districts of Sierra Leone. Findings from this study will be valuable for expanding and sustaining the triple elimination initiative in Sierra Leone and similar contexts, particularly within the African region.

## METHODS

The study population consisted of pregnant women accessing ANC services at selected public facilities in four TES districts of Sierra Leone (Western Area Urban [WAU], Bombali, Bo, and Kenema) between 1 November 2024 and 31 October 2025.

This study employs a cross-sectional design that examines secondary data on HIV, syphilis, and HepB testing among pregnant women, which is routinely collected in the elimination of mother-to-child transmission (EMTCT) facility registers.

### General setting

Sierra Leone is a country in West Africa with a population of approximately 8 million,^21^ with women of childbearing age (15–49) accounting for 22.2% of the total population.^22^

The country is divided administratively into 4 regions and 16 districts. Health care services are delivered through a tiered system comprising primary, secondary, and tertiary facilities. A network of peripheral health units forms the backbone of primary health care, providing essential services to communities, including ANC services for pregnant women. Hospitals are responsible for more advanced care at the secondary and tertiary levels. For this study, 21 health facilities (HFs) were selected across four districts, with each district representing one of Sierra Leone’s four regions to ensure national representation of triple elimination testing. HFs were purposively selected based on availability of integrated triple testing, routine provision of ANC services, adequate client volume, and geographic representation of Sierra Leone’s four regions. The selected HFs routinely provide integrated screening for HIV, syphilis, and HepB to pregnant women during their first ANC visit. Only HFs offering all three tests were included. Sites lacking triple testing capacity were excluded to ensure consistent evaluation of service delivery and programme performance across locations.

### Specific settings

Testing for HIV, Syphilis, and HepB at ANCs is carried out by trained nurse counsellors using the WHO prequalified rapid diagnostic test (RDT) kits. For HIV and Syphilis, a single duo test kit is used, followed by HIV-determine RDT kits to confirm the positive HIV results from the duo test kits. For HepB, a separate Determine-Early HBsAg RDT is used without other confirmatory testing for positive results. Nurse counsellors at various HFs nationwide are responsible for completing these registers. Every month, data from these HFs, except for HepB test data, are summarised and submitted to the district health information system-2 (DHIS2) database.

### Data collection and sources

Data for this study were abstracted from the EMTCT facility registers by a team of trained final year medical students using the Epicollect5 electronic tool designed specifically for this study. Data from November 2024 to June 2025 were collected in July 2025, and monthly thereafter until the end of the study period. Additionally, we used the DHIS2 database to calculate individual district screening coverage and prevalence for HIV and Syphilis (routinely reported into the DHIS2) during the same study period, to compare the findings with those of our study (Table 3).

### Data management and statistical analysis

The data were exported into Microsoft Excel spreadsheet, disaggregated by HF level (primary, secondary, and tertiary) and by districts. Before analysis was carried out, data validation was done by the principal investigator and other staff of the Disease Prevention and Control Directorate, according to the MoH standard procedures for routine data quality assessment. This included checks for completeness, consistency, and accuracy. Analysis was done using STATA version 16 (StataCorp, College Station, TX, USA), and the primary outcomes of interest were the screening coverage and the test positivity of each of the infections. The screening coverage was calculated using the number of women screened for each infection out of all those who attended their first ANC visit, stratified by district and by HF level. We calculated the positivity of each of the three infections by dividing the number of individuals who tested positive by the total number of individuals screened, stratified by district and HF level. Data were analysed descriptively using frequencies and proportions along with their 95% confidence interval as appropriate.

### Ethical statement

Ethics approval for the study was obtained from the Sierra Leone Ethics and Scientific Review Committee (SLESRC-025/02/2025). Permission to use the data from the EMTCT register was obtained from the Chief Medical Officer of the MoH. As secondary anonymised data were used, the issue of informed consent did not apply. Data used for this study were stripped of personal identifiers, and only aggregate data were presented. The electronic databases were kept on a password-protected computer of the principal investigator.

## RESULTS

A total of 10,003 pregnant women attended their first ANC visit at selected facilities during the study period. Screening coverage was high across all infections, with 99.6% tested for HIV, 99.6% for syphilis, and 92.9% for HepB (Figure). The positivity rates of HIV, HepB, and syphilis were 4.9%, 4.0%, and 1.3%, respectively. Thirty-three (0.3%) women were positive for both HIV and syphilis, and another 33 (0.4%) were positive for both HepB and syphilis. Fifty-six women (0.6%) were co-infected with HIV and HepB. Eleven women (0.1%) were found to be positive for all three infections (Table 1).

**FIGURE.**
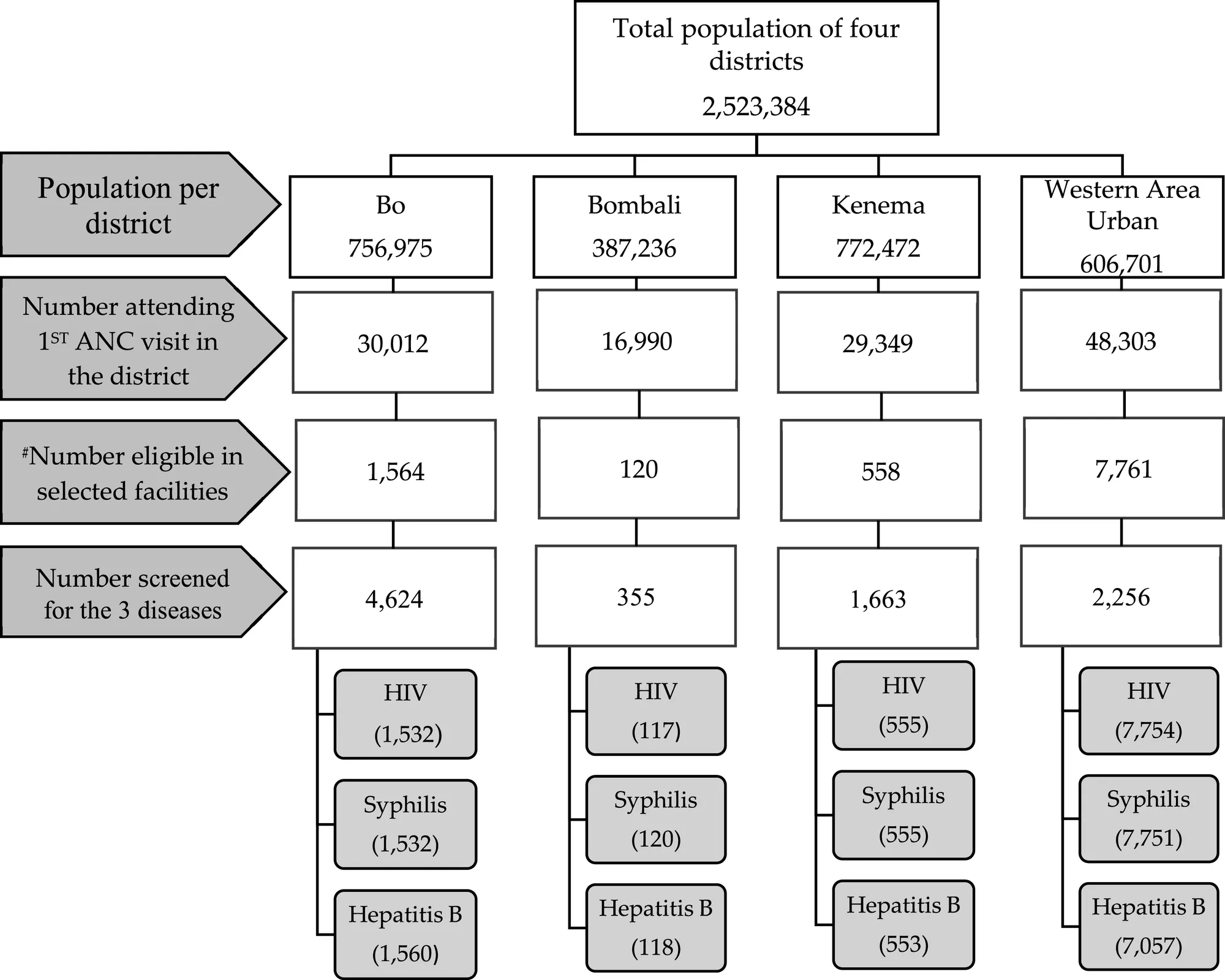
Summary of the population per district, number of pregnant women eligible, and the number of pregnant women tested for HIV, syphilis, and hepatitis B at first antenatal care (ANC) visits in selected facilities across four districts in Sierra Leone, between November 2024 and October 2025. This figure presents testing counts by district; their positivity rates are reported in Table 1.

**TABLE 1.** Co-infections among pregnant women who tested positive for HIV, hepatitis B, or syphilis at their first antenatal care visit in selected facilities, Sierra Leone, November 2024 to October 2025.

Co-infection	Number screened	Positivity n (%)^A^
HIV–HepB	9,250	56 (0.60)
HIV–Syphilis	9,948	33 (0.33)
HepB–Syphilis	9,251	33 (0.36)
HIV–Syphilis–HepB	9,240	11 (0.10)

A Row percentage of total tested positive (numerator) and total tested (denominator).

### District-level screening coverage and positivity

Across districts, screening coverage exceeded 97%. WAU recorded a high positivity rate for both HIV (5.0%) and HepB (4.8%). In Bo District, HIV positivity rate was 4.1%, HepB 1.1%, and syphilis 0.2%, while Kenema showed a lower HIV positivity rate (2.2%) with very low positivity for syphilis and no HepB infection found. Bombali reported an exceptionally high HIV, syphilis, and HepB positivity (19.7%, 48.3%, and 16.3%, respectively) and the greatest burden of HIV–syphilis co-infection (0.3%), HIV–HepB co-infection (11.3%), HepB–syphilis co-infection (0.3%), and HIV–syphilis–HepB co-infection. Co-infection rate for all three infections is 0.1%, also reported in Bombali (Table 2).

**TABLE 2.** District-level screening coverage and positivity of HIV, Syphilis, and Hepatitis B infection among pregnant women attending ANC clinics in Sierra Leone between November 2024 and October 2025.

District		HIV		Hepatitis B		Syphilis	
	Total eligible^A^ Number	Screened	Positive	Screened	Positive	Screened	Positive
		N (%)^B^	N (%)^C^	N (%)^B^	N (%)^C^	N (%)^B^	N (%)^C^
Bo	1,564	1,532 (98)	62 (4.1)	1,560 (99.7)	17 (1.1)	1,532 (98)	3 (0.2)
Kenema	558	555 (99)	12 (2.2)	553 (99.1)	00	555 (99.5)	3 (0.5)
Bombali	120	117 (97)	23 (19.7)	118 (98.3)	20 (16.3)	120 (100)	58 (48.3)
Western Area Urban	7,761	7,754 (99)	387 (5.0)	7,057 (91.0)	338 (4.8)	7,751 (99.9)	67 (0.9)
Total	10,003	9,958	484	9,288	375	9,958	131
Total positivity		484 (4.9%, 95% Cl: 4.44–5.30)		375 (4.0%, 95% Cl: 3.6–4.5)		131 (1.3%, 95% Cl: 1.1–1.6)	
		HIV–HBV Co-infection. 56 out of 9,250^D^ (0.6%, 95% Cl: 0.5–0.8)				–	–

ANC = antenatal care; CI = confidence interval.

A Number eligible for screening is the number of women who had their first antenatal care clinic visit during the study period.

B Percentage calculated with number eligible as the denominator.

C Percentage calculated with number screened as denominator.

D 9,250 women were tested for HIV and hepatitis.

### Facility-level screening coverage and positivity

Screening coverage remained above 97% across all tiers: 97.8% in primary, 99.8% in secondary, and 99.8% in tertiary facilities. HIV positivity rate was highest in secondary facilities (6.7%), followed by tertiary (4.3%) and primary (4.0%) levels. HepB positivity also peaked in secondary facilities (6.0%), while syphilis positivity reached 3.1% at the same level. HIV–HepB co-infection occurred only in secondary (1.1%) and tertiary (0.5%) facilities (Table 3).

**TABLE 3.** Screening coverage and positivity of HIV, Syphilis, and Hepatitis B infection among pregnant women attending ANC clinics at different levels of health facilities (Primary, Secondary, and Tertiary) in Sierra Leone between November 2024 and October 2025.

Level	Number eligible^A^	HIV				Syphilis				Hepatitis B			
		Screened		Positive		Screened		Positive		Screened		Positive	
	N	N	(%)^B^	N	(%)^C^	N	(%)^B^	N	(%)^C^	N	(%)^B^	N	(%)^C^
Primary	1,466	1,434	(97.8%)	56	(4.0%)	1,434	(97.8%)	2	(0.1%)	1,462	(99.7%)	13	(0.9%)
Secondary	2,555	2,550	(99.8%)	170	(6.7%)	2,553	(99.9%)	78	(3.1%)	2,553	99.9%)	152	(6.0%)
Tertiary	5,982	5,974	(99.8%)	258	(4.3%)	5,972	(99.8%)	51	(0.9%)	5,276	(88.2%)	210	(4.0%)

ANC = antenatal care.

A Number eligible for screening is the number of women who had their first antenatal care clinic visit during the study period.

B Percentage calculated with number eligible as denominator.

C Percentage calculated with number screened as denominator.

### Routine testing for HIV and syphilis first ANC visits in the four target districts captured in the DHIS2

Testing coverage for the two infections was reported as lower than the coverage of the triple testing approach. The HIV prevalence in the total target populations of Bo, Kenema, WAU, and Bombali were found to be 2%, 1%, 3%, and 1%, respectively. The syphilis prevalence in the total target populations of Bo, Kenema, WAU, and Bombali were 1%, 0%, 8%, and 3%, respectively (Table 4).

**TABLE 4.** Testing coverage and prevalence of HIV and syphilis among pregnant women during their first ANC visit reported in the DHIS2 across the four districts between November 2024 and October 2025.

		HIV				Syphilis			
Districts	Number of antenatal clients at first visit^A^	Total tested for HIV	Total tested positive for HIV	HIV testing rate^B^	HIV prevalence \^C^	Total tested for syphilis	Total tested positive for syphilis	Syphilis testing rate^B^	Syphilis prevalence^C^
Bombali District	17,466	16,438	198	94%	1%	14,793	387	85%	3%
Bo District	30,963	22,853	503	74%	2%	26,403	227	85%	1%
Kenema District	30,971	22,831	247	74%	1%	25,022	10	81%	0%
Western Area Urban District	49,682	39,387	1,293	79%	3%	43,352	3,441	87%	8%

ANC = antenatal care.

A Number eligible for screening is the number of women who had their first antenatal care clinic visit during the study period.

B Row percentage of total tested (numerator) and Number of antenatal clients at first visit (denominator).

C Row percentage of total tested positive (numerator) and total tested (denominator).

## DISCUSSION

This is the first study to describe the screening coverage and positivity rate of triple testing for HIV, syphilis, and HepB in Sierra Leone. There are two major findings. First, screening coverage was high for all infections, with 99.6% tested for HIV, 99.6% for syphilis, and 92.9% for HepB. This meets the 90% service-delivery benchmarks for the TES. Lower HepB screening coverage reflects the recent, phased introduction of HepB testing within ANC services, compared with long-established HIV and syphilis programmes, rather than test stock-outs or service refusal. This coverage was higher than that reported in the DHIS2 for all routine HIV and syphilis testing done for first ANC attendees in the districts. The high level of uptake demonstrates the feasibility of integrated screening of the three infections in ANC, even in low-resource settings, aligning with WHO guidance.^23^ This achievement, partly due to the availability of testing kits from the TES supported by the Global Fund, should be sustained and expanded to ensure a continuum of care for all pregnant women. Although EMTCT services for HIV and syphilis are well established in Sierra Leone, our findings indicate persistent gaps in access and continuity of prevention and treatment services. Strengthening uptake of existing EMTCT interventions remains essential and should be implemented in parallel with HepB prevention to maximise maternal and child-health outcomes. Studies from Sierra Leone and other countries have documented suboptimal access to treatment and prevention services, even with higher coverage of HepB screening.^24,25^ This indicates missed opportunities for integrated care, necessitating improved care services in a well-coordinated approach. While tuberculosis and malaria screening were beyond the scope of the current TES implementation, future integration should be considered where diagnostic pathways, resources, and service readiness allow. Second, the positivity rates for HIV, HepB, and syphilis were 4.9%, 4.0%, and 1.3%, respectively, and co-infections were not uncommon, to the pooled HIV–HBV co-infection prevalence of 3.3% among pregnant women in sub-Saharan Africa,^26^ and the co-infection rate (0.6%) in this study is lower than some West-African estimates (∼5.2%).^23^

This study was not designed as a prevalence survey. Nevertheless, the HIV and syphilis positivity rates were similar to the prevalence of the two infections as determined by the routine testing reported in the DHIS2, at least in Bo, Kenema, and WAU. For these three districts, we suggest that our HepB test positivity rate may be close to what a prevalence survey would find. Bombali district, however, was an outlier with markedly high HIV (19.7%), syphilis (48.3%), and HepB (16.3%) positivity rates found in our study. It is unlikely that these positivity rates in Bombali reflect the prevalence in ANC first-visit attendees in the district, since the HIV and syphilis rates are so much higher than the prevalence determined through routine testing (obtained through DHIS2). Only 120 women were reported as eligible for the three diseases at selected HFs in Bombali out of a total district-wide ANC first-attendee population of 16,990 (0.7%). For comparison, the numbers of eligible women reported in Bo, Kenema, and WAU study facilities were 1,564 (5.2%), 558 (1.9%), and 7,761 (16.1%), respectively. The atypically high positivity rates observed in Bombali are unlikely to reflect district-wide prevalence and more plausibly indicate selective testing, protocol deviations, or localised epidemiological clustering. These challenges underscore the importance of standardised implementation, supervision, and routine data quality assurance during programme scale-up.

The results of our study indicate that the prevalence of HepB in ANC populations in Sierra Leone may be lower than in some other African countries.^15^ However, even a prevalence of around 4% would be associated with a significant burden of disease when applied to such a large ANC population. As an example, WAU district alone has an annual first-visit ANC population of 48,303, of which 1,449 pregnant women would be infected with HepB. Without effective intervention, rates of MTCT of HepB exceed 90%.^17,27^ Assuming all pregnancies resulted in live births, over 1,300 children would be infected. These stark figures underscore the urgent need for measures to prevent MTCT of HepB. While interventions for prevention of HIV and syphilis MTCT are well-established in Sierra Leone, preventive interventions for HepB MTCT are yet to be introduced. Aside from testing, the TES framework recommends maternal treatment, safe delivery, infant follow-up, and vaccination.^17,28^ There is an urgent need to fast-track the implementation of Sierra Leone’s TES using a person-centred life-course approach encompassing integrated screening, treatment, follow-up, and prevention for pregnant women, infants, and their partners across antenatal, delivery, postnatal, and child-care platforms.

The strengths of this study rely on the sample size, selection of different tiers of HFs with triple testing capability and regional headquarters towns, which are likely to be reflective of the contextual situation regionally. Additionally, data collection carried out by trained medical students and the validation process by the national team limit systematic biases that may be associated with the data. Finally, we adhered to the STROBE (Strengthening the Reporting of Observational Studies in Epidemiology) guidelines for the conduct and reporting of observational studies.^29^ Apparent limitations included reliance on routine programme data, which may be subject to recording errors or missing data; potential under-detection of infections (especially for HBV, where only surface antigen was assessed); and generalisability restricted to four districts.

## CONCLUSION

The high screening coverage demonstrates the operational feasibility of TES. There are disparities in positivity of the three diseases and their co-infection across districts. Expanding and sustaining triple elimination testing is crucial for the continuous care of pregnant women. Urgent implementation of Sierra Leone’s TES with a person-centred life-stage approach focusing on preventive measures for HepB among pregnant women is needed to address the significant disease burden.

## References

[1] Wu S, Prevalence of human immunodeficiency virus, syphilis, and hepatitis B and C virus infections in pregnant women: a systematic review and meta-analysis. Clin Microbiol Infect. 2023;29(8):1000-1007. 36921717 10.1016/j.cmi.2023.03.002

[2] Dionne-Odom J, Hepatitis B, HIV, and syphilis seroprevalence in pregnant women and blood donors in Cameroon. Infect Dis Obstet Gynecol. 2016;2016(1):4359401-4359408. 27578957 10.1155/2016/4359401 PMC4992796

[3] Cohn J, Eliminating mother-to-child transmission of human immunodeficiency virus, syphilis and hepatitis B in sub-Saharan Africa. Bull World Health Organ. 2021;99(4):287-295. 33953446 10.2471/BLT.20.272559 PMC8085625

[4] Yah CS, Tambo E. Why is mother to child transmission (MTCT) of HIV a continual threat to new-borns in sub-Saharan Africa (SSA). J Infect Public Health. 2019;12(2):213-223. 30415979 10.1016/j.jiph.2018.10.008

[5] World Health Organization. Global guidance on criteria and processes for validation: elimination of mother-to-child transmission of HIV and syphilis. Geneva: WHO, 2014.

[6] Zhang L, Integrated approach for triple elimination of mother-to-child transmission of HIV, hepatitis B and syphilis is highly effective and cost-effective: an economic evaluation. Int J Epidemiol. 2019;48(4):1327-1339. 30879066 10.1093/ije/dyz037

[7] Woodring J, Integrating HIV, hepatitis B and syphilis screening and treatment through the maternal, newborn and child health platform to reach global elimination targets. Western Pac Surveill Response J. 2017;8(4):1-5. 10.5365/wpsar.2017.8.3.005 PMC5803553 29487757

[8] Wang AL, Integrated prevention of mother-to-child transmission for human immunodeficiency virus, syphilis and hepatitis B virus in China. Bull World Health Organ. 2015;93(1):52-56. 25558108 10.2471/BLT.14.139626 PMC4271682

[9] Hawkes SJ, Gomez GB, Broutet N. Early antenatal care: does it make a difference to outcomes of pregnancy associated with syphilis? A systematic review and meta-analysis. PLoS One. 2013;8(2):e56713. 23468875 10.1371/journal.pone.0056713 PMC3585307

[10] Nelson NP, Jamieson DJ, Murphy TV. Prevention of perinatal hepatitis B virus transmission. J Pediatr Infect Dis Soc. 2014;3(suppl_1):S7–S12. 10.1093/jpids/piu064 PMC4164184 25232477

[11] Le STT, Antenatal maternal hepatitis B care is a predictor of timely perinatal administration of hepatitis B immunoglobulin. Intern Med J. 2017;47(8):915-922. 28444819 10.1111/imj.13466

[12] Raviglione M, Maher D. Ending infectious diseases in the era of the sustainable development goals. Porto Biomed J. 2017;2(5):140-142. 32258607 10.1016/j.pbj.2017.08.001 PMC6806806

[13] Ward JW, Hinman AR. What is needed to eliminate hepatitis B virus and hepatitis C virus as global health threats. Gastroenterology. 2019;156(2):297-310. 30391470 10.1053/j.gastro.2018.10.048

[14] Visser M, Evaluating progress towards triple elimination of mother-to-child transmission of HIV, syphilis and hepatitis B in The Netherlands. BMC Public Health. 2019;19(1):353. 30922277 10.1186/s12889-019-6668-6 PMC6440074

[15] Azhali BA, Setiabudi D, Alam A. Evaluating the impact of triple elimination program for mother-to-child transmission of HIV, syphilis, and hepatitis B in Indonesia. Narra J. 2023;3(3):e405. 38455604 10.52225/narra.v3i3.405 PMC10919734

[16] Roy P, Dasgupta R. Making sense of the triple elimination initiative against mother-to-child transmission of HIV, syphilis, and hepatitis B in the Indian context. Indian J Public Health. 2024;68(1):1-2. 38847624 10.4103/ijph.ijph_307_24

[17] Bell L, Progress towards triple elimination of mother-to-child transmission of HIV, hepatitis B and syphilis in Pacific Island Countries and Territories: a systematic review. Lancet Reg Health West Pac. 2023;35:100740. 37424691 10.1016/j.lanwpc.2023.100740 PMC10326693

[18] Yendewa GA, Prevalence of hepatitis B surface antigen and serological markers of other endemic infections in HIV-infected children, adolescents and pregnant women in Sierra Leone: a cross-sectional study. Int J Infect Dis. 2021;102:45-52. 33002619 10.1016/j.ijid.2020.09.1459

[19] Jiba DF, Sero-prevalence of syphilis infection among people living with HIV in Sierra Leone: a cross-sectional nationwide hospital-based study. BMC Infect Dis. 2023;23(1):762. 37932713 10.1186/s12879-023-08740-9 PMC10626761

[20] Djibo DA, Prevalence and risk Factors for human immunodeficiency virus (HIV) and syphilis infections among military personnel in Sierra Leone. Curr HIV Res. 2017;15(2):128-136. 28521722 10.2174/1570162X15666170517101349

[21] Odia O. 2021 Mid-Term Population and Housing Census - December 10, 2021. Stats SL. 2022. https://www.statistics.sl/index.php/census/mid-term-population-census.html.

[22] Statistics Sierra Leone (Stats SL). Sierra Leone demographic and health survey 2019. Freetown, Sierra Leone: Statistics Sierra Leone (Stats SL), 2020. https://dhsprogram.com/publications/publication-FR365-DHS-Final-Reports.cfm.

[23] Harrison J, Rapid systematic review of interventions to improve antenatal screening rates for syphilis, hepatitis B, and HIV in low- and middle-income countries. Int J Gynecol Obstet. 2024;166(1):3-26. 10.1002/ijgo.15425 38391190

[24] Kazibwe A, Facilitators, barriers and service availability for delivering integrated care for the triple elimination of HIV, syphilis and hepatitis B vertical transmission in Uganda: a multi-site explanatory mixed methods study. BMC Health Serv Res. 2025;25(1):626. 40307877 10.1186/s12913-025-12797-4 PMC12044932

[25] Mustapha M, Evaluation of Sierra Leone’s elimination of mother-to-child transmission of HIV program, 2024: the need for a life stages approach to triple elimination. PLOS Glob Public Health. 2026;6(4):e0005491. 42048344 10.1371/journal.pgph.0005491 PMC13123933

[26] Platt L, Prevalence and burden of HBV co-infection among people living with HIV: a global systematic review and meta-analysis. J Viral Hepat. 2020;27(3):294-315. 31603999 10.1111/jvh.13217 PMC7383613

[27] Veronese P, Prevention of vertical transmission of hepatitis B virus infection. World J Gastroenterol. 2021;27(26):4182-4193. 34326618 10.3748/wjg.v27.i26.4182 PMC8311536

[28] World Health Organization. Introducing a framework for implementing triple elimination of mother-to-child transmission of HIV, syphilis and hepatitis B virus: policy brief. Geneva: WHO, 2024.

[29] von Elm E, The Strengthening the Reporting of Observational Studies in Epidemiology (STROBE) statement: guidelines for reporting observational studies. J Clin Epidemiol. 2008;61(4):344-349. 18313558 10.1016/j.jclinepi.2007.11.008

